# Prevalence of malaria and its risk factors in Lake Tana and surrounding areas, northwest Ethiopia

**DOI:** 10.1186/s12936-022-04310-7

**Published:** 2022-11-04

**Authors:** Fasil Adugna, Melaku Wale, Endalkachew Nibret

**Affiliations:** 1 Department of Biology, Bahir Dar University, 79, Bahir Dar, Ethiopia; 2 Biotechnology Research Institute, Bahir Dar University, Bahir Dar, Ethiopia

**Keywords:** Prevalence, Malaria, Risk factor, *Plasmodium* infection, Ethiopia

## Abstract

**Background:**

In Ethiopia, malaria is a major concern to the health, and socio-economic development of the country because of its occurrence at the peak agricultural activities. Factors such as environmental, human host, parasite, and vector determine malaria transmission. Therefore, the present study was conducted to determine the prevalence and associated factors of malaria among febrile patients who visited selected health centres.

**Methods:**

Institutional-based cross-sectional study was conducted between October 2020 to July 2021 in eight selected health centres located in Lake Tana and its surrounding areas. A simple random sampling technique was used to select febrile patients. Thick and thin blood films were prepared and processed according to the WHO guidelines. Socio-demographic and malaria risk factors were collected from study participants who could read and write using a self-administered questionnaire, whereas face-to-face interview was used to collect information from those participants who could not write and read. The strength of association between risk factors and malaria was assessed using univariate and multivariate logistic regression models.

**Results:**

Of the total (531) febrile patients, 75.3% were malaria negative and 24.7% (overall prevalence) were malaria confirmed cases. Most of the infections were caused by *Plasmodium falciparum* (72.5%) followed by *Plasmodium vivax* (23.7%) and mixed-species (3.8%). The highest prevalence was recorded in Kidist Hana (51.5%) followed by Robit (34.8%), Gorgora (30.3%), and Wusha Tiris (25%) health centres. In terms of months, the highest prevalence (37.5%) was detected in October whereas the lowest (14%) was in March. Logistic regression analysis revealed that gender (p = 0.023), educational level (p = 0.025), study month (p = 0.036), presence of eave in the house (p = 0.002) and wall openings (p = 0.041), not using bed nets (p = 0.001), sleeping in the same house with cattle (p = 0.031) and the distance between mosquito-breeding site and living house (p = 0.020) were explanatory risk factors significantly associated with malaria among studied participants.

**Conclusions:**

In this study, we confirmed that the occurrence of malaria prevalence was high and continued against the Ethiopian malaria elimination plan of 2021–2025. Therefore, to meet the goals of this plan, the current prevention and control efforts should be stepped up even better in the coming years.

## Background

Mosquito-borne diseases are the world’s major causes of illness and death, particularly in tropical and subtropical countries [1]. Among these, malaria infection is one of the leading public health problems in the world. Malaria is caused by protozoan parasites that belong to the genus *Plasmodium* [2, 3], that are transmitted to humans via the bite of infected female *Anopheles* mosquito [2, 4]. Among these, the four species: *Plasmodium falciparum, Plasmodium vivax, Plasmodium ovale,* and *Plasmodium malariae* are known to infect human beings in Ethiopia [5]. From the four *Plasmodium* species, *P. falciparum* is more severe than others in terms of morbidity and mortality, followed by *P. vivax* [6] with proportions of 60% and 40%, respectively [7].

Globally, nearly half of the population lives in areas at risk of malaria transmission [4]. Children below five years and pregnant women are among the most susceptible groups. After successful declines were recorded for two decades (2000–2015), the rate of reduction of malaria mortality and morbidity were decreased in the period 2016–2018 compared to the period 2010–2015 [8]. In addition, regions known to be free of malaria began to report cases of malaria indicating that the disease is expanding probably in response to global warming [9]. The latest estimates of the World Health Organization (WHO) showed 241 million new cases and 627,000 deaths in the world. Most of these malaria cases and deaths were in the WHO African regions (94%), followed by South-East Asia (3%) [10]. Therefore, malaria still remains a major public health problem affecting many countries in the world [11, 12]. Clinical complications and manifestations observed in malaria include nervous involvement, respiratory distress, renal failure, metabolic acidosis, and hypoglycaemia [13].

Most malaria cases and death occur in sub-Saharan Africa (SSA) [11, 14, 15]. It carries the bulk of the global malaria burden, with the highest global cases and deaths [8, 16, 17]. Ethiopia is one of the sub-Saharan African countries with malaria morbidity and mortality. It is a major concern in the country, and it can cause much damage to the health and socioeconomic development of the country due to the occurrence of malaria during harvesting seasons which reduces agricultural productivity and hence leads to food insecurity and poverty [18]. About 75% of the landmass of Ethiopia is considered malarious and approximately 68% (54 million) of the Ethiopian population lives in malaria-risk areas [14, 19]. The threats of malaria cases are concentrated in the western low lands of Oromia, Amhara, Tigray and nearly the entire Regional State of Gambella, and Benishangul Gumuz Regional State [20]. The major epidemics occur cyclically every 5–8 years in Ethiopia, but focal epidemics are occurring every year [21]. About 2.9 million cases of malaria and 4782 related deaths have been reported annually, and the rate of morbidity and mortality dramatically increases during epidemics [19, 22].

The distribution and transmission pattern of malaria in Ethiopia differ from place to place depending on climate, rainfall patterns, and altitude [17, 23]. The transmission of malaria is generally unstable and seasonal [24]. In Ethiopia, there are two malaria transmission periods, the first one is the main transmission period that occurs between September and December (following the rain from June to August), and the second occurs between April and June (due to the February and March rains) [14, 25–28].

In Ethiopia, malaria control strategies are very complex and influenced by various factors. Among these strategies, indoor residual spraying (IRS) and long-lasting insecticidal nets (LLINs) are the most important malaria vector control strategies [29]. Additionally, the introduction of the rapid diagnostic tests at the community level and adaptation of artemisinin-based combination therapy (ACT) for malaria-infected individuals, are also practiced in Ethiopia [30]. However, insecticide resistance on IRS, and LLINs in different parts of the country affect the control of major malaria vectors. In Ethiopia, the development of resistance to different insecticide groups by *Anopheles arabiensis* was reported [29, 31, 32].

The transmission and incidence rate of malaria infection is determined by different factors such as environmental, human host, parasite, and vector [8, 27]. In addition to these, the targeted malaria intervention requires the proper identification of factors influencing malaria risk in the community [33]. In Ethiopia, several factors, socio-demographic, place of study, house conditions, breeding site of mosquitoes, malaria control and prevention practices, human sleeping behavior and knowledge and practice of people about malaria have been identified as risk factors for malaria infection [34–40].

Malaria has continued to be one of the major public health problems in Amhara Regional State. It accounts for 31% of Ethiopia’s malaria burden [41]. In 2012, a total of 1,127,241 malaria cases were recorded within the region. Among eleven zones in the region, only five of them found in West Amhara accounted for 93.1% of the total malaria burden. West Gojjam Zone reported the greatest number of cases followed by North Gondar (former division), South Gondar, and Awi [42].

The weekly reports of each district health office indicated that many people who lived in and around Lake Tana suffered from malaria. This is due to the presence of suitable altitude, temperature and habitats for breeding and development of malaria vectors in Lake Tana and its surrounding localities. The present study was aimed to assess the prevalence and associated factors of malaria among febrile patients visiting eight selected health centres in Lake Tana and surrounding areas.

## Methods

### Study area description

This prospective malaria prevalence study was conducted in different locations of Lake Tana and its surrounding areas. Lake Tana is the source of the Blue Nile and it is the largest lake in Ethiopia, which contributes up to 60% of the Nile’s water and 50% of the country’s freshwater. The lake is located in Amhara Regional State at latitude of 11°36' N and a longitude of 37°23' E. The major portion of the total annual rainfall is observed between June and September. The minimum mean temperatures of the study area is 10.9 °C and the maximum is 29.2 °C [43]. Lake Tana contains thirty-seven islands and peninsulas dispersed all over the lake [44]. Twenty of these islands shelter churches and monasteries of significant historical, cultural, and religious interest. Lake Tana is rich in biodiversity including several species of fish, amphibians, macro-invertebrates, and micro-invertebrates. Because of its richness in biodiversity, the United Nations Educational, Scientific, and Cultural Organization (UNESCO) recognized Lake Tana as a Biosphere Reserve in 2015 [45].

**Fig. 1:**
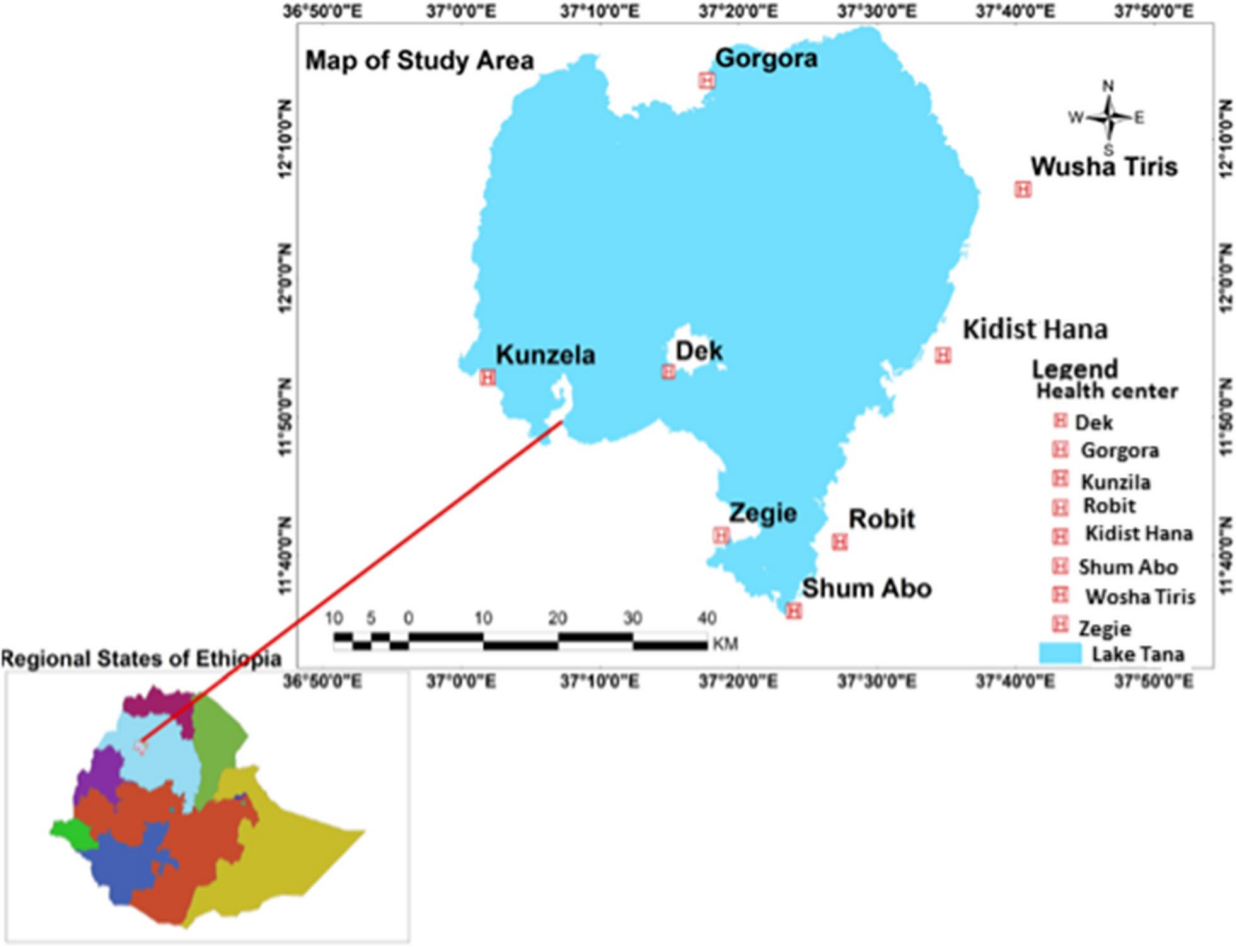
Map of the study area showing the study sites and eight health centres around Lake Tana, northwest Ethiopia

Islands, peninsulas and mainland areas are found in Lake Tana scattered all over the five districts, i.e., Semein Achefer, Bahir Dar Zuria, Fogera, Libokemkem and Chuahit and one city (Bahir Dar city administration), and these were selected purposively based on accessibility, suitability and proximity of areas to the local inhabitants. The data were collected from one island (Dek), two peninsulas (Gorgora, and Zegie), and five surrounding mainland areas (Kunzila, Shum Abo, Robit, Kidist Hana and Wusha Tiris) (Fig. 1).

### Study design and period

An institutional-based cross-sectional study was employed to determine the current malaria prevalence in eight purposively selected health centres. The clinical data were collected for ten consecutive months from October 2020 to July 2021.

### Source and target population

The source population of the study was all patients coming to selected health centres for treatment during the study periods. All symptomatic or febrile patients visiting the governmental health institutions for treatment during the study period were considered as a target population.

### Inclusion and exclusion criteria

All symptomatic or febrile patients (body temperature above 37.5 °C) visiting the selected health centres during the study period and willing to participate were included in the study. Individuals taking anti-malarial drugs or those who took anti-malarial drugs for three weeks before the study period were excluded.

### Sample size determination

The sample size was calculated using a single population proportion formula. The prevalence of malaria among the general population (38.3%) in Bahir Dar town health centres was used to determine the sample size [46]. The sample size was calculated as follows using the single population proportion formula: n = (Z_α/2_)^2^ *p*(1-p)/d^2^, where: n = the total sample size, P = prevalence of malaria in Bahir Dar town health centres (38.3%), Zα/2 is the critical value of normal distribution at 95% CI (1.96), d = the desired precision of the estimate/margin of error (5%). After adding 5% to compensate for the non-responses and 1.5-design effect, the total sample size was 571. This sample size was allocated in each health centre based on the number of populations residing in the catchment areas.

### Sampling technique and study variables

The Zones, districts, Kebeles (the smallest administrative unit) and health centres were selected purposively based on accessibility, suitability and proximity of areas to the local inhabitants. However, study participants (symptomatic or febrile patients) coming to the health centres for blood film examination were selected using a simple random sampling method to reduce sampling bias during sampling. This is happen due to each member of the population has an exactly equal probability of being chosen in a basic random sampling process. The sample was collected monthly until the final sample size was reached. Dependent variable consist of malaria prevalence whereas the independent variable consists of socio-economic characteristics and other malaria-associated risk factors.

### Methods of data collection

The following data collection methods were used to accomplish this study.

#### Questionnaire-based data collection

The socio-demographic and other malaria risk factors were collected using pre-tested structured questionnaires. For those study participants who had difficulty in reading and filling out the questionnaires, face-to-face interviews, based on questionnaire, were made by trained laboratory technicians. A self-administered questionnaire was used for adult study participants aged above 18 years who were capable of reading whereas a face-to-face interview was made with parents/guardians of children.

#### Blood sample collection and processing

Finger-prick blood samples were collected using a strictly disposable sterile blood lancet. Thick and thin blood films were prepared for the purpose of checking the presence of *Plasmodium* parasites and species identification, respectively, according to WHO guidelines [47]. All blood films were stained with 3% Giemsa and examined by two experienced laboratory technicians independently for the presence of *Plasmodium* infection and species identification. If there was controversy on the results observed by the two, a third person resolved the disagreement. Microscopy or Rapid Diagnostic Test (RDT, Access Bio Korea, Inc, Korea, Seoul) was used for diagnosis of study participants.

### Ethics considerations

Before data collection, an ethics clearance letter was received from the Ethics Clearance Committee of the College of Science, Bahir Dar University with Ref. No. PRCSVD/08/2020. A permission letter or supportive letter was obtained from Amhara Public Health Institute. The objective, potential risks and benefits of the study were explained in detail for health centre heads, adult febrile patients and their parents/guardians. Questionnaire-based data and blood sample collection were made after obtaining written consent from adult febrile patients and parents/guardians of children under 18 years of age.

### Data analysis

Data was entered and analysed using SPSS 20. Descriptive statistics were used to describe the prevalence, frequency and proportion of study participants in connection to different risk factors. The association of malaria prevalence and risk factors were assessed using univariate and multivariate logistic regression statistical models. In univariate logistic regression analysis, those variables with less than 0.25 p-values were entered into a multivariate logistic regression model [48]. The strength of association between malaria occurrence and associated factors were measured using Crude Odds Ratio (COR) and Adjusted Odds Ratio (AOR). A 95% confidence interval and p-value less than 0.05 were used to declare statistically significant risk factors associated with malaria.

## Results

### Socio-demographic characteristics of study participants

The data for this malaria prevalence was gathered from different health centres located in Lake Tana and its surrounding areas. In the study period, a total of 531 febrile patients were included and most of them were diagnosed using a microscope (n = 473; 89.1%). Males, teenagers and middle-aged people, widowed ones, rural residents, private occupations, farmers, and students were more prone to infection. Infection levels looked similar among education levels of patients and religions. Infection levels were less on patients who earned less income. RDT tested patients were found more prone to be positive than microscope examination (Table 1).

**Table 1:** Univariate and multivariate logistic regression analysis of risk factors associated with malaria prevalence among febrile patients attending selected health centres

Variables	Malaria status		Total (%)	COR (95% Cl)	P-value	AOR	P-value
	Positive cases (%)	Negative cases (%)					
Socio-demographic variables							
Gender							
Male	95 (29)	233 (71)	328 (61.8)	1.89 (1.23–2.91)	0.004*	57.5 (1.75–1888.3)	0.023*
Female	36 (17.7)	167 (82.3)	203 (38.2)	1	–	1	–
Age in years							
< 5	6 (20.7)	23 (79.3)	29 (5.5)	1.13 (0.39–3.26)	0.821	–	–
5–19	50 (28.9)	123 (71.1)	173 (32.6)	1.76 (0.92–3.74)	0.088	–	–
20–44	60 (24.1)	189 (75.9)	249 (46.9)	1.38 (0.73–2.5)	0.323	–	–
> 44	15 (19)	65 (81)	80 (15.1)	1	–	–	–-
Marital status							
Single	65 (25.9)	186 (74.1)	251 (47.3)	1	–	–	–
Married	63 (23.7)	203 (76.3)	266 (50)	0.89 (0.60–1.32)	0.560	–	–
Divorced	1 (11.1)	8 (88.9)	9 (1.7)	0.36 (0.04–2.92)	0.337	–	–
Widowed	2 (40)	3 (60)	5 (1)	1.91(0.31–11.67)	0.485	–	–
Residence							
Town	19 (15)	108 (85)	127 (23.9)	1	–	–	–
Rural	112 (27.7)	292 (72.3)	404 (76.1)	2.18 (1.28–3.72)	0.004*	–	–
Occupation							
Government	7 (18.9)	30 (81.1)	37 (7)	1	–	–	–
Private	8 (27.6)	21 (72.4)	29 (5.5)	1.63 (0.51–5.19)	0.407	–	–
Merchant	3 (14.3)	18 (85.7)	21 (4)	0.71 (0.16–3.12)	0.654	–	–
Labourer	2 (18.2)	9 (81.8)	11 (2.1)	0.95 (0.17–5.42)	0.956	–-	–
Farmer	55 (27.4)	146 (72.6)	201 (37.8)	1.61 (0.67–3.89)	0.286	–	–
Student	42 (25.9)	120 (74.1)	162 (30.5)	1.50 (0.61–3.67)	0.374	–	–
Housewife	14 (20)	56 (80)	70 (13.2)	1.07 (0.39–2.94)	0.893	–	–
Educational level							
College and above	8 (17)	39 (83)	47 (8.8)	1	–	1	–
Secondary (9–12)	13 (21.7)	47 (78.3)	60 (11.3)	1.35 (0.51–3.58	0.549	–	–
Elementary (1–8)	42 (27.3)	112 (72.7)	154 (29)	1.83 (0.79–4.23)	0.159	0.00 (0.00–0.35)	0.025*
Pre-school(Kg1-3)	1 (25)	3 (75)	4 (0.7)	1.62(0.15–17.69)	0.690	–	–
Read and write	19 (22.3)	66 (77.7)	85 (16)	1.40 (0.56–3.51)	0.468	–	–
Illiterate	48 (26.5)	133 (73.5)	181 (34.1)	1.76 (0.77–4.03)	0.182	–	–
Family monthly income (ETB)							
< 2280	17 (15)	96 (85)	113 (21.3)	0.38 (0.30–1.54)	0.353	–	–
2280–3040	74 (27.8)	192 (72.2)	266 (50.1)	1.48 (0.74–2.94)	0.267	–	–
3040–4560	28 (29.8)	66 (70.2)	94 (17.7)	1.63 (0.75–3.57)	0.218	–	–
> 4560	12 (20.7)	46 (79.3)	58 (10.9)	1	–	–	–
Name of health centres							
Health centres							
Kunzila	7 (10.4)	60 (89.6)	67 (12.6)	0.35 (0.13–0.92)	0.033*	–	–
Dek	9 (13.6)	57 (86.4)	66 (12.4)	0.47 (0.19–1.17)	0.105	–	–
Zegie	12 (17.6)	56 (82.4)	68 (12.8)	0.64 (0.28–1.49)	0.304	–	–
Shum Abo	10 (14.4)	58 (85.3)	68 (12.8)	0.52 (0.21–1.24)	0.141	–	–
Robit	23 (34.8)	43 (65.2)	66 (12.4)	1.61 (0.75–3.43)	0.222	–	–
Kidiste Hana	34 (51.5)	32 (48.5)	66 (12.4)	3.19 (1.52–6.71	0.002*	–	–
Gorgora	20 (30.3)	46 (69.7)	66 (12.4)	1.30 (0.60–2.82)	0.500	–	–
Wusha Tirs	16 (25)	48 (75)	64 (12.1)	1	–	–	–
Months of the study							
Months							
March	7 (14)	43 (86)	50 (9.4)	1	–	1	–
November	17 (30.3)	39 (69.7)	56 (10.5)	2.68 (1.00–7.14)	0.049*	–	–
December	14 (27.4)	37 (72.6)	51 (9.6)	2.32 (0.85–6.37)	0.101	–	–
January	11 (20.4)	43 (89.6)	54 (10.2)	1.57 (0.56–4.44)	0.393	–	–
February	7 (14.3)	42 (85.7)	49 (9.2)	1.02 (0.33–3.17)	0.967	–	–
October	21 (37.5)	35 (62.5)	56 (10.5)	3.69 (1.41–9.67)	0.008*	104.4 (1.35–8096.7)	0.036*
April	9 (18.4)	40 (81.6)	49 (9.2)	1.38 (0.47–4.06)	0.556	–	–
May	15 (27.8)	39 (72.2)	54 (10.2)	2.36 (0.87–6.40)	0.091	–	–
June	17 (30.3)	39 (69.7)	56 (10.5)	2.68 (1.00–7.14)	0.049*	–	–
July	13 (23.2)	43 (76.8)	56 (10.5)	1.86 (0.67–5.11)	0.230	–	–
House conditions							
House roof type							
Thatched	1 (33.3)	2 (66.7)	3 (0.6)	1.53(0.14–17.02)	0.729	–	–
Corrugated iron	130 (24.6)	398 (75.4)	528 (99.4)	1	–	–	–
House wall types							
Mud plastered	128 (25.1)	382 (74.9)	510 (96)	2.01 (0.44–9.10)	0.365	–	–
Stone wall	1 (14.3)	6 (85.7)	7 (1.3)	1.00(0.08–13.37)	1.000	–	–
Brick wall	2 (14.3)	12 (85.7)	14 (2.6)	1	–	–	–
Eave opening							
Yes	104 (42.1)	143 (57.9)	247 (46.5)	6.92(4.33–11.08)	0.000*	100.4 (5.35–1884.0)	0.002*
No	27 (9.5)	257 (90.5)	284 (53.5)	1	–	1	–
Wall opening							
Yes	69 (27.7)	180 (72.3)	249 (46.9)	1.36 (0.92–2.02)	0.127	19.5 (1.13–339.5)	0.041*
No	62 (22)	220 (78)	282 (53.1)	1	–-	1	–
Malaria prevention practices and behavior							
Use of bed net in home							
Yes	43 (33.1)	87 (66.9)	130 (24.5)	1	–	–	–
No	88 (21.9)	313 (78.1)	401 (75.5)	3.22 (2.08–4.98)	0.000*	–	–
Types of bed net							
Impregnated	37 (21)	139 (79)	176 (33.1)	1	–	–	–
None	31 (29.8)	73 (71.1)	104 (19.6)	1.32 (0.63–2.77)	0.458	–	–
Family members who use bed net							
Whole family	13 (7.5)	161 (92.5)	174 (32.8)	1	–	1	–
Few	55 (51.9)	51 (48.1)	106 (20)	14.64(5.74–37.3)	0.000*	489.2 (11.31–21167.5)	0.001*
Chemical Spray							
Yes	3 (33.3)	6 (66.7)	9 (1.7)	1	–	–	–
No	128 (24.5)	394 (75.5)	522 (98.3)	0.65 (0.16–2.63)	0.546	–	–
Outdoor Sleeping							
Yes	43 (33.1)	87 (66.9)	130 (24.5)	1.76 (1.14–2.72)	0.011*	–	–
No	88 (21.9)	313 (78.1)	401 (75.5)	1	–	–	–
Outdoor activities							
Yes	82 (28.9)	202 (71.1)	284 (53.5)	1.64 (1.09–2.46)	0.016*	–	–
No	49 (19.8)	198 (80.2)	247 (45.9)	1	–	–	–
Cattle in the house							
Yes	63 (34.4)	120 (65.6)	183 (34.5)	2.16 (1.44–3.24)	0.000*	48.0 (1.42–1624.7)	0.031*
No	68 (19.5)	280 (80.5)	348 (65.5)	1	–	1	–
Mosquito breeding site distance from the house							
Breeding area distance							
< 1 km	63 (42.3)	86 (57.7)	149 (28.1)	6.19 (3.44–11.12)	0.000*	222.9 (2.35–21106.3)	0.020*
1–2 km	50 (23.6)	162 (76.4)	212 (39.9)	2.61 (1.46–4.67)	0.001*	1	–
> 2 km	18 (10.6)	152 (89.4)	170 (32)	1	–	–	–
Previous malaria infection and diagnostic methods							
Previous infection							
Yes	41(27.1)	110 (72.9)	151 (28.4)	1.20 (0.78–1.85)	0.403	–	–
No	90 (23.7)	290 (76.3)	380 (71.6)	1	–	–	–

* Significant association

### Malaria cases across study health centres and months

**Fig. 2:**
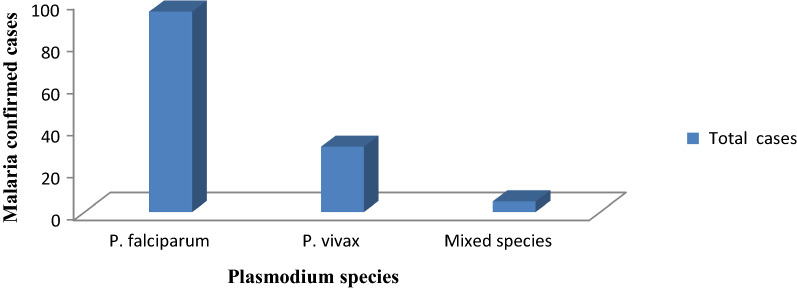
A number of confirmed malaria cases and *Plasmodium* parasites detected in selected health centres around Lake Tana, northwest Ethiopia, 2021

**Fig. 3:**
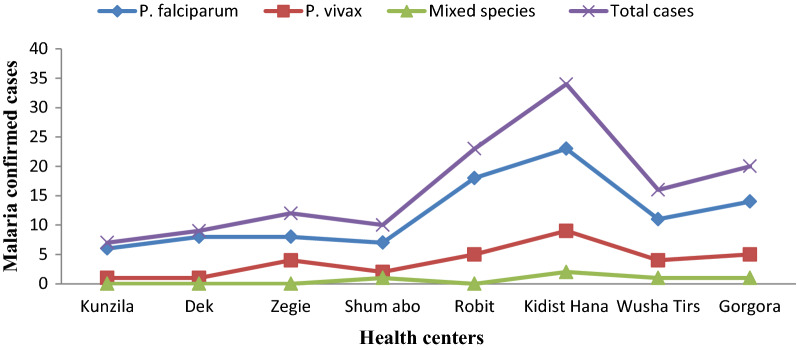
Number of malaria confirmed cases and *Plasmodium* species detected in and around Lake Tana, northwest Ethiopia, over ten-month period in 2021

**Fig. 4:**
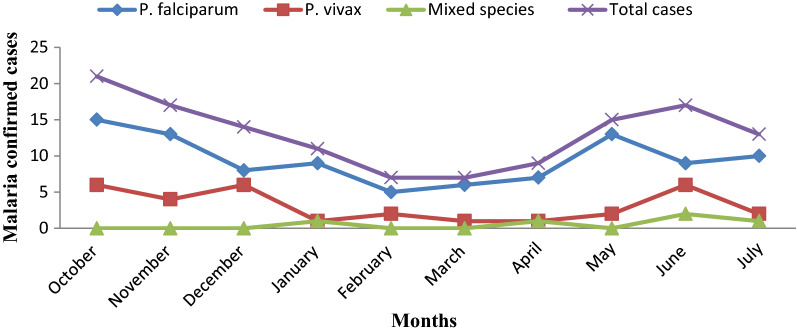
Monthly malaria confirmed cases and *Plasmodium* species detected in selected health centres in and around Lake Tana, northwest Ethiopia, 2021

Out of 531 febrile patients suspected of malaria, 131 were confirmed to be malaria cases. From the total 131 malaria confirmed cases, *P. falciparum* accounted for 95 (72.5%), *P. vivax* for 31 (23.7%), and mixed-species (*P. falciparum* and *P. vivax*) for 5 (3.8%) (Fig. 2). The highest malaria case was recorded in Kidist Hana health centre (n = 34; 26%). *Plasmodium falciparum* and *P. vivax* were recorded in all health centres and months, but mixed-species were recorded only at Shum Abo, Kidisit Hana, Wusha Tirs and Gorgora health centres, and in the month of January, April, June and July (Figs. 3 and 4). The highest number of case was observed in October followed by November and June (Fig. 4).

### Malaria prevalence in the study area

**Fig. 5:**
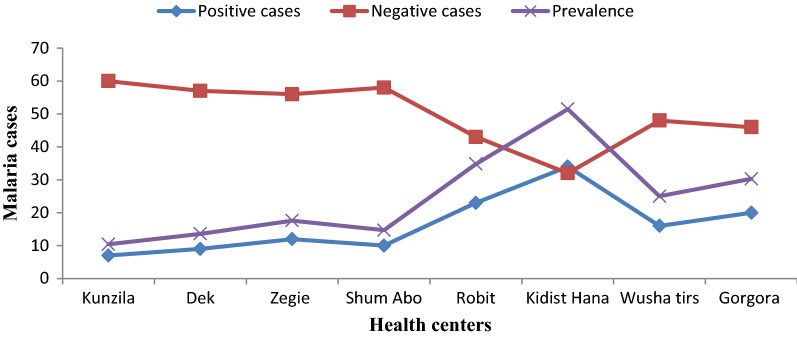
Total confirmed and suspected malaria cases in selected health centres around Lake Tana, northwest Ethiopia, 2021

**Fig. 6:**
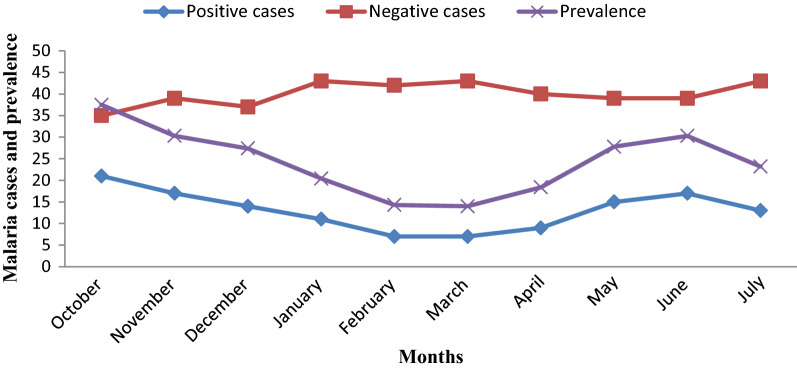
Total confirmed and suspected malaria cases in study months in and around Lake Tana, northwest Ethiopia, 2021

Of the total (531) malaria suspected patients, 400 (75.3%) were malaria negative and 131 (24.7%) were malaria confirmed cases. The overall prevalence was 24.7%. Out of this, 17.9% were accounted for by *P. falciparum.* The prevalence of malaria was highly variable among different health centres, ranging from 10.4 to 51.5% (p < 0*.*001). The highest prevalence was recorded in Kidist Hana (51.5%) followed by Robit (34.8%), Gorgora (30.3%), and Wusha Tiris (25%) health centres (Fig. 5). In terms of months, the highest prevalence (37.5%) was observed in October whereas the lowest (14%) was observed in March (p = 0.036) (Fig. 6).

### Multivariate logistic regression analysis for selected risk factors for malaria

A multivariate logistic model was used to analsze risk factors that had p < 0.25 in the univariate logistic regression analysis result (Table 1). Only eight risk factors appeared in the final multivariate logistic model with p < 0.05 and were found to be independent explanatory risk factors for malaria among studied participants in the study area. Male study participants were more likely to be infected with *Plasmodium* parasites than females (AOR = 57.5, 95% CI 1.75–1888.3, p = 0.023). The probability of malaria infection was 100 times higher in patients who had eave openings in their houses than in those who had not eave openings in their houses (AOR = 100.4, 95% CI 5.35–1884.0, p = 0.002). Similarly, all family members who did not use bed nets at night were at higher risk of malaria compared with those family members who used bed nets (AOR = 489.2, 95% CI 11.31–21167.5, p = 0.001). Additionally, participants who live within < 1 km distance of mosquito breeding site had a 223-fold higher probability of *Plasmodium* infection than those who lived > 2 km (AOR = 222.9, 95% CI 2.35–21106.3, p = 0.021). Furthermore, the result of this analysis showed that elementary school education level (p = 0.025), living in the study areas in the month of October (p = 0.036), staying with cattle all night in the house (p = 0.031), and presence of wall openings in the houses were significantly associated with malaria.

## Discussion

In Ethiopia, malaria is a major concern and it can cause much damage to the health, and socio-economic development of the country due to the occurrence of malaria during harvesting seasons [18]. The result of this study revealed that the overall malaria prevalence was 24.7%. This prevalence is higher than other similar findings reported in different parts of Ethiopia, for example from Dilla town and the surrounding rural areas [35], East Shewa Zone of Oromia Regional State [36], Arba Minch Hospital [49], Jimma town [34], Ziway health centre [50], Ethiopian malaria survey [51], and Oromia Regional State [52]. Similarly, it is greater than pooled malaria prevalence of pregnant women (12.72%) [53] and adults (13.61%) [54] conducted in Ethiopia. The result is consistent among studies conducted in different parts of Ethiopia, such as Hadiya Zone of Southern Ethiopia [38], pooled prevalence (25.8%) of malaria in Ethiopia [55], and East Shewa Zone of Oromia Regional State [40]. On the other hand, it is lower than similar studies carried out in Haro Limmu district of Western Ethiopia [37], district of Northwest Ethiopia [39], Awash, Metehara and Ziway areas [56], Bahir Dar Health centres [46] and Hallaba health centre of southern Ethiopia [57]. This variation might be due to the differences in breeding sites for the *Anopheles* mosquito, altitudinal differences, and microclimate variations. Moreover, the reasons might be a lifestyle change, geographical area, communities awareness difference, development of dams or irrigation, economic status, and the type of malaria diagnosis methods used [58].

The relatively lower malaria prevalence in the current study might be connected to malaria eradication and control programs, such as the use of long-lasting insecticide-treated nets (LLIN), insecticide residual spray (IRS), the introduction of rapid diagnostic tests, and the use of artemisinin-based combination therapies, might have led to a decrease in a load of malaria [30]. Furthermore, it was a combined prevalence of different health centres, which is carried out in different areas of Lake Tana and surrounding localities.

Malaria prevalence was more predominant in the Kidist Hana health centre (51.5%) compared to other health centres (p = 0.002). However, the least malaria prevalence was recorded in Kunzila (p = 0.033). This dissimilarity is due to community awareness differences about the use of malaria intervention activities, geographical location, altitude difference, and temperature [59]. In addition to this, the presence or absence of flooding due to expansions of Lake Tana during the rainy season, which favours breeding of mosquitoes results in relatively more or fewer malaria cases.

In this study, the predominant *Plasmodium* parasite detected was *P. falciparum (*72.5%), followed *P. vivax* (23.7%) and mixed-species (3.8%). This result is in line with other previous studies carried out in different parts of Ethiopia [21, 51, 60–62]. On the other hand, the finding of the study is quite different from the previous reports in which *P. falciparum* proportion is much lower than those of *P. vivax* in Ethiopia [63, 64]. This discrepancy might be due to the difference in the study area, study period, local climate, malaria control, and prevention methods and laboratory capabilities [63]. Additionally, this variation might be due to differences in topographic differences and intrinsic factors of the parasites [65]. In contrast, the molecular study conducted at the North Gondar zone confirmed the presence of *P. ovale* [66].

In multivariate analysis, gender of study participants, elementary school education level, living in the study areas in the month of October, the presence of eave and wall openings in the house, family members who use bed nets, staying with cattle all night in the house and mosquito breeding site distance from the house were significantly associated with malaria prevalence. On the other hand, age, income, sleep under bed nets, outdoor sleeping, outdoor activity, residence area, and sleeping with cattle assumed to be associated factors in the univariate analysis did not consider as explanatory factors in multivariate analysis.

The current study showed males (29%) were more susceptible to malaria than females (17.7%) (p = 0.004). The finding of this study is similar to studies carried out in different parts of Ethiopia [60, 64, 65, 67–69]. This difference might be due to males were often engaged in early night outdoor agricultural activities, hence, having a higher chance of exposure to infected mosquitoes. Another study showed that larger mosquito human-biting activities occur outdoors than indoors and during the early parts of the night, indicating higher outdoor malaria transmission potential in Ethiopia [70].

Elementary school study participants were more likely to be infected with *Plasmodium* infection than other participants who joined college and above educational level (p = 0.025). Educational level was significantly associated with malaria prevalence in studies carried out in different parts of Ethiopia [37, 71, 72]. This could be justified by increased awareness of the well-educated individuals to proper use of bed nets during sleeping which increases protection of malaria among the whole families. On the other hand, other studies carried out in Ethiopia that indicated educational level was not a significant factor for malaria transmission [34, 73].

In this study, the highest malaria prevalence was reported in October (37.5%) whereas the lowest was in March (14%) (p = 0.036). This is due to October is one of the major malaria transmission seasons that occurs following the rain from June to August [26, 27]. This study is in agreement with similar studies reported in Felegehiwot Referral Hospital [68], Tselemti district [60], East Shewa Zone of Oromia Regional State [40], and East Wollega Zone [37].

In Ethiopia, environmental management is the key component of vector control among the national malaria prevention and control strategies [74]. In this study, individuals living around a short distance of a stagnant water site had a significantly increased risk of having malaria than those who had no such site. This is due to the fact that these areas are suitable for the breeding of mosquitoes and they can cause more mosquito bites. This finding is agreed with studies done in different parts of Ethiopia, such as Dilla Town and the surrounding rural areas [35], Jimma town [34] and Dembia district [73].

Among Ethiopian malaria control strategies, long-lasting insecticidal nets (LLINs) are the most important malaria vector control strategies [29, 74, 75]. All members of the family who always slept under mosquito net were less likely to be infected with malaria parasite than those who did not use bed net during sleeping time (p = 0.001). This is in agreement with studies done in different parts of Ethiopia [35, 36, 38, 39, 73]. This result is contradicted by some studies carried out in some African countries showing the use of bed nets did not indicate a significant difference in malaria cases and deaths [34, 76]. In addition, house conditions with eave and wall openings had shown the presence of significant associations with malaria prevalence which is similar to other study in Ethiopia [37]. The risk of exposure to mosquito biting could be high in houses having eave and wall openings because these allow mosquitoes to have access into the house and increase the probability of their contact with their hosts. In contrast, a similar study conducted in Gondar town showed house conditions had no significant association with malaria prevalence [77].

The result of this study showed sleeping in the same house with cattle was the main determinant factor for malaria (p = 0.031). This finding is in agreement with other similar study conducted in Malawi [78]. The availability of alternative hosts such as livestock near or inside residential houses could make suitable mosquito breeding sites as well as could attract more mosquitoes towards the house. Therefore, the chance of human hosts to receiving more mosquito bites and infection would be high. This finding is opposed to similar studies carried out in Dembia district [73].

## Conclusion

The current study showed malaria prevalence was much greater than the pooled prevalence of malaria among adults and pregnant women in Ethiopia as well as it is continued to go against the Ethiopian malaria elimination plan of 2021–2025. Gender of study participants, elementary school education level, living in the study areas in the month of October, the presence of eave, and wall openings in the houses, family members who did not use a bed net, staying with cattle all night in the house and mosquito breeding site distance from the houses were confirmed to be the risk factors for malaria prevalence. Hence, to avoid these problems, the current prevention and control methods such as health education, environmental management practice, larviciding, use of LLIN and application of IRS for controlling the vector, prompt diagnosis and treatment, and use of ACT should be effectively implemented in the study area.

## Data Availability

The data used and analyzed in this study is available from this manuscript.
